# Thirty years after German reunification: population health between solidarity and global competitiveness

**DOI:** 10.1093/eurpub/ckaa119

**Published:** 2020-10-05

**Authors:** Rok Hrzic, Helmut Brand

**Affiliations:** c1 Department of International Health, Care and Public Health Research Institute – CAPHRI, Maastricht University, The Netherlands; c2 Prasanna School of Public Health, Manipal Academy of Higher Education, Manipal, India

The reunification of Germany was not planned. The sudden economic and political crisis of the German Democratic Republic (East Germany) in 1989 surprised political elites on both sides of the Iron Curtain. In a matter of weeks, the East German communist regime was dismantled. To avoid a complete financial collapse of the newly democratic East Germany, the two sides first established a monetary, economic and social union by the early summer of 1990. The political reunification was complete by October. The makeshift nature of the process is apparent from the legal mechanism used, Article 23 of the West German Basic Law, under which the new eastern German states joined the Federal Republic by fully adopting its laws and institutions. It sidestepped Article 146 of the Basic Law, which would have created a union between the two states under a new constitution, and would have entailed a more comprehensive evaluation of the East German institutions.

Thus, the reunification of Germany was an act of grand political improvization. But it was also a radical experiment in solidarity, aiming to create parity in living conditions between the new and the old Federal states. The tools that were to engineer this outcome were the Solidarity tax—the ‘Soli’—and the Solidarity Pact. The first of these tools, a surcharge on personal income, capital gains and corporate income, was designed to generate additional income for the Federal government, and to help pay for the second of these tools, a funding scheme that would rebuild the eastern German states. After three decades, this experiment is over. The Solidarity pact expired at the end of 2019; and, after sustained public pressure, the Bundestag passed a law that will abolish the Soli for most taxpayers as of 2021.^1^

The reunification of the country improved health in East Germany. By 2008, life expectancy in the region had increased by an estimated 5.7 years for men and 4 years for women.^2^ Today, East German women enjoy a slightly higher life expectancy than women in the West, while the life expectancy of East German men lags a year behind that of West German men. It is clear, however, that the health benefits of German reunification were not equally distributed. While East Germans aged 60 or older saw substantial mortality benefits after reunification, the economic restructuring and downturn that followed reunification hit working-age East Germans hard, and damaged their health.^3^

We have yet to learn everything the German reunification experiment has to teach us. There are, for example, important regional differences in how reunification has affected life expectancy trajectories.^4^ It seems that in the better-off urban centres of eastern Germany, the policies that were implemented following reunification led to important health improvements, even as the health of people living in rural and deindustrialized areas continued to stagnate or worsened under the new pressures of global competition. Today in Germany, the north–south and the urban–rural health disparities overshadow the east–west divide. For example, the inhabitants of the eastern city of Dresden are healthier today than the population of the western Ruhr area.

Behind these new regional disparities lies a fundamental question about the role of regional development in the global marketplace. Today, Germany competes with places like Silicon Valley and Shenzhen. The dilemma is clear: Is it better to funnel resources generated by Daimler, Porsche and Bosch in Baden-Württemberg back into the region to support its competitiveness, or should these resources be used to support struggling areas by the Baltic Sea in order to raise their standard of living and improve their health outcomes? Solidarity is not only in direct conflict with global competitiveness; it has repeatedly lost out to competitiveness in our era of economized politics.

Reunified Germany is a microcosm of European integration. Many Central and Eastern European countries followed the path of East Germany from behind the Iron Curtain to embrace Western political and economic structures. They joined the European Union (EU), which required them to adopt its law and institutions. The EU also faces the same difficult trade-off between solidarity and global competitiveness. The austerity debacle in the aftermath of the Great Recession demonstrated that EU policy prioritized economic competition with the USA and China over solidarity with the struggling Member States. EU leaders seem to have learned from this painful experience and are responding to the economic consequences of the COVID-19 pandemic with proposals that recognize the importance of solidarity.^5^ It is clear that the lessons of German reunification are highly relevant to many questions the EU is facing after its eastern expansion, including how to engineer a convergence of health between the new and the old Member States. In dealing with these challenges, we should take full advantage of the scientific opportunity this unique, three-decade long stretch of German history offers the European public health community.

*Conflicts of interest*: None declared. 
